# Meningeal lymphatic architecture and drainage dynamics surrounding the human middle meningeal artery

**DOI:** 10.1016/j.isci.2025.113693

**Published:** 2025-10-04

**Authors:** Mehmet Albayram, Sutton B. Richmond, Kaan Yagmurlu, Ibrahim S. Tuna, Eda Karakaya, Hiranmayi Ravichandran, Fatih Tufan, Emal Lesha, Melike Mut, Filiz Bunyak, Yashar.S. Kalani, Adviye Ergul, Rachael D. Seidler, Onder Albayram

**Affiliations:** 1 Department of Radiology, Adventhealth, Orlando, FL, USA; 2 Department of Radiology, University of Florida College of Medicine, Gainesville, FL, USA; 3 Brain Rehabilitation Research Center, Malcom Randall VA Medical Center, Gainesville, FL, USA; 4 The University of Tennessee Health Science Center, Department of Neurosurgery, Memphis, Tenn, USA; 5 Department of Pathology and Laboratory Medicine, Medical University of South Carolina, Charleston, SC, USA; 6 Ralph H. Jackson Department of Veterans Affairs Medical Center, Charleston, SC, USA; 7 Department of Physiology and Biophysics, Institute for Computational Biomedicine, Weill Cornell Medicine, New York, NY, USA; 8 Caryl and Israel Englander Institute for Precision Medicine, Weill Cornell Medicine, New York, NY, USA; 9 Istanbul Esenler Avicenna Hospital, Department of Internal Medicine and Geriatrics, Istanbul, Turkiye; 10 Department of Neurosurgery, University of Virginia, Charlottesville, VA, USA; 11 Department of Electrical Engineering and Computer Science, University of Missouri, Columbia, MO, USA; 12 St. John’s Neuroscience Institute, Oklahoma State University, School of Medicine, Tulsa, OK, USA; 13 Department of Applied Physiology and Kinesiology, University of Florida, Gainesville, FL, USA; 14 Norman Fixel Institute for Neurological Diseases, University of Florida, Gainesville, FL, USA; 15 Department of Neuroscience, Medical University of South Carolina, Charleston, SC, USA

**Keywords:** Anatomy, Neuroscience, Vascular anatomy

## Abstract

The anatomical basis of cerebrospinal and interstitial fluid clearance along the human ventral meninges remains poorly defined. Here, we present the *in vivo* and *ex vivo* evidence of a distinct CSF-ISF drainage compartment surrounding the middle meningeal artery (MMA) in humans. In five healthy participants, dynamic contrast-enhanced MRI revealed delayed signal enhancement along the MMA-peripheral region, peaking at 90 min, substantially later than adjacent vascular and lymphatic structures, consistent with slower, nonvascular clearance dynamics. To validate these findings, we performed the high-resolution spatial mapping of the dorsal and ventral dura using immunofluorescence and imaging mass cytometry, identifying PROX1+, PDPN+, and LYVE1+ lymphatic-like structures that are aligned along the MMA. These findings define a previously unrecognized outflow zone in the human ventral dura and support the presence of organized meningeal lymphatic architecture beyond dorsal compartments. This integrated human framework motivates future mechanistic studies into the role of ventral lymphatics in brain fluid clearance and neuroimmune regulation.

## Highlights

• Human MRI shows delayed CSF-ISF clearance along the ventral of the middle meningeal artery
• The dural layer near the MMA shows distinct spatiotemporal kinetics from the vascular pathway
• A rich, compartmentalized lymphatic network is in the ventral dura via dual-modality mapping
• This study provides an integrated human evidence of ventral meningeal outflow pathways

## Graphical abstract

**Figure:**
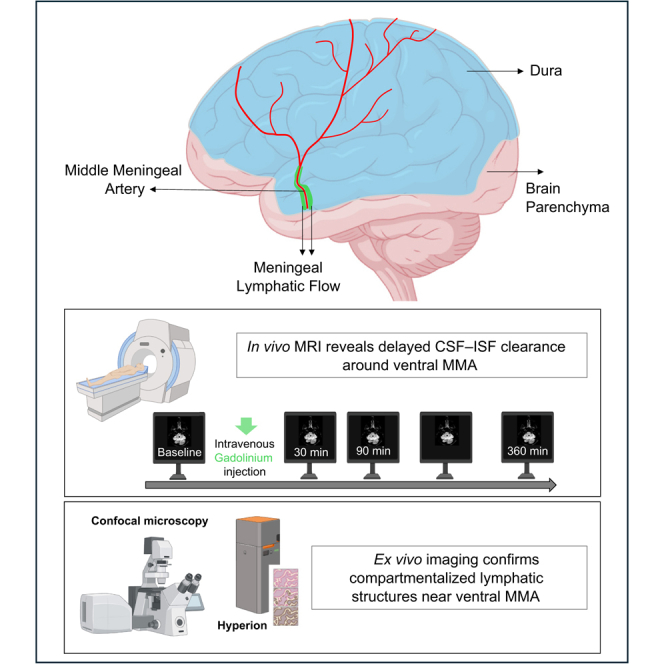

## Introduction

Over the past decade, the rediscovery of lymphatic vessels within the meninges has fundamentally reshaped our understanding of cerebrospinal fluid (CSF) dynamics and immune surveillance in the central nervous system (CNS).^1^^,^^2^ These meningeal lymphatic vessels (MLVs), located predominantly in the dura mater, form a critical interface between the CNS and peripheral lymphatic circulation. Initially characterized in rodent models, MLVs have been shown to facilitate the clearance of CSF, interstitial fluid (ISF), macromolecules, and immune cells from the subarachnoid space to the deep cervical lymph nodes.^1^^,^^2^^,^^3^ Their identification has challenged the long-standing view of the brain as an immune-privileged organ and introduced a framework for understanding neuroimmune interactions and disease pathogenesis.

Most early studies have focused on dorsal meningeal lymphatics, particularly those lining the superior sagittal and transverse sinuses.^1^^,^^3^^,^^4^^,^^5^ These structures have been extensively mapped using both tracer-based approaches in rodents and advanced imaging in humans and non-human primates.^4^^,^^6^ More recent work in animal models has expanded attention to lateral and ventral cranial compartments, including the petrosquamous, sigmoid, and cavernous sinuses, as well as regions adjacent to the pterygopalatine artery and middle meningeal artery (MMA).^3^^,^^7^ These regions have become a focus of interest for their potential involvement in CSF and ISF clearance, though their role in the human brain is still not well understood.

In parallel, the development of *in vivo* imaging modalities, particularly contrast-enhanced MRI and real-time tracer tracking, has provided insights into functional CSF-ISF pathways. While most imaging studies to date have captured dorsal lymphatic outflow, there is growing interest in the ventral skull base, including the MMA and adjacent fossae, as potential drainage zones with distinct physiological roles. If active in the human brain, these ventral pathways could play a central role in fluid homeostasis, immune trafficking, and clearance of metabolic waste—functions that are particularly vulnerable in aging, neurodegeneration, and traumatic brain injury.

In this study, we examined CSF-ISF drainage and associated meningeal lymphatic activity in the ventral dura surrounding the MMA of healthy human participants. Using dynamic intravenous contrast-enhanced MRI, we identified a previously unrecognized CSF-ISF transfer pattern along the ventral MMA, distinct from adjacent vascular and lymphoid structures. To anatomically validate this finding, we performed high-resolution histological analyses of dorsal and ventral MMA-associated meninges using two orthogonal modalities: immunofluorescence confocal microscopy and Hyperion™ Imaging Mass Cytometry (IMC). These methods allowed the detailed visualization of lymphatic structures and spatial compartmentalization across the dural layers.

To our knowledge, this is a comprehensive study to characterize ventral meningeal lymphatic architecture and CSF-ISF flow patterns in the healthy human brain. We identify a distinct lymphatic network adjacent to the MMA and propose that this region functions as a previously unrecognized ventral outflow hub in CNS fluid clearance. These findings expand the anatomical and functional atlas of human MLVs, laying the groundwork for future studies of ventral drainage dynamics in both health and disease.

## Results

### Spatiotemporal MRI mapping identifies delayed ventral drainage around the human middle meningeal artery

**Figure 1:**
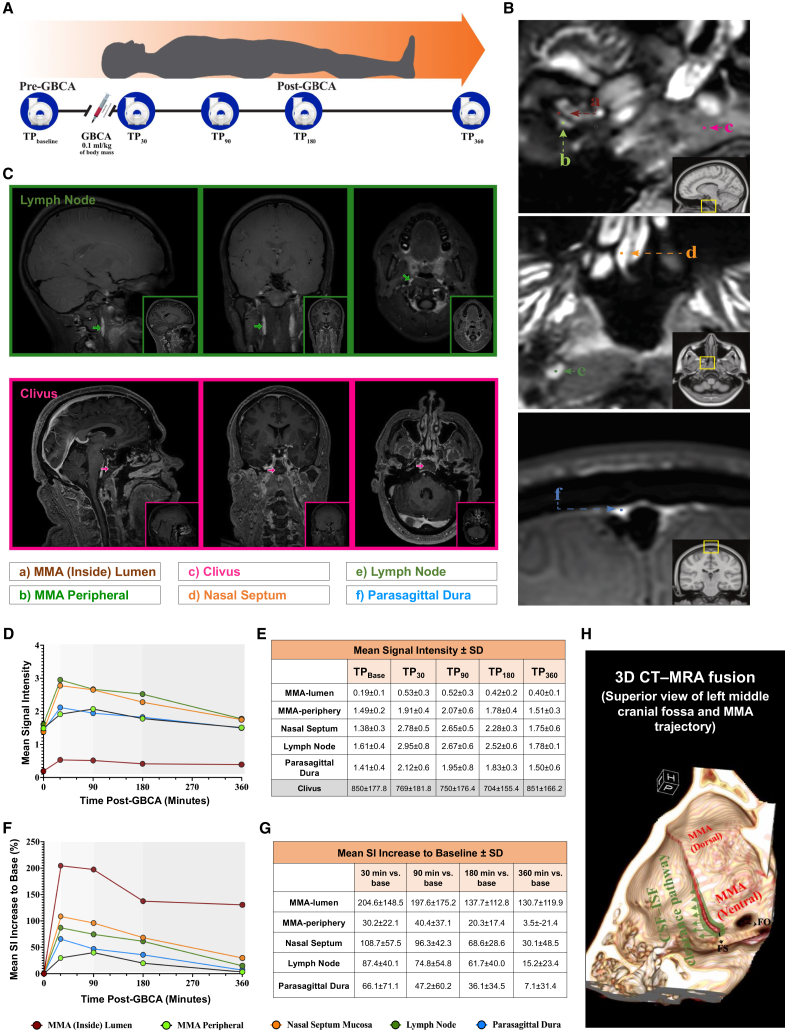
Dynamic contrast-enhanced MRI reveals delayed CSF-ISF drainage dynamics around the MMA in healthy human participants (A) Schematic of the imaging timeline. Each participant underwent five MRI acquisitions over a 6-h period: one pre-contrast baseline scan (TP_baseline_) and four post-contrast scans at 30 (TP_30_), 90 (TP_90_), 180 (TP_180_), and 360 (TP_360_) minutes. (B) Locations of six spherical regions of interest (ROIs; diameter = 0.7 mm): a) MMA lumen, b) MMA periphery, c) Clivus (used for signal normalization), d) Nasal septum mucosa, e) Cervical lymph node, and f) Parasagittal dura. ROIs were placed consistently across participants (*n* = 5) by two board-certified neuroradiologists. (C) The 3D multi-plane MRI panel includes sagittal, axial, and coronal views, specifically illustrating the location of the deep cervical lymph node (green) and clivus (pink). (D) Time-course plots of normalized mean signal intensity (SI) for each ROI (normalized to the clivus). Most ROIs demonstrated an early post-contrast SI rise followed by a gradual return toward baseline. The MMA periphery showed a more prolonged SI elevation, with delayed decline after TP_90_. (E) Descriptive metrics for the normalized (to Clivus ROI values) SI mean (± standard deviation) at each time point for the five ROIs. This table also includes the reported Clivus mean SI values (± standard deviation), and serves as the quantitative reference for panel D. (F) Percent change in SI relative to baseline (TP_baseline_) for each ROI. While most structures peaked at TP_30_, the MMA periphery showed a sustained increase in SI through TP_90_. (G) Descriptive statistics at each time point for the normalized changes in SI relative to baseline (expressed as a percent change) at each ROI location. Between-participant variance is high; therefore, error bars and confidence intervals have been omitted. See panel G for detailed mean ± standard deviation values corresponding to panel (F). (H) Schematic diagram depicts a hypothetical CSF–ISF clearance route along the ventral dura surrounding the MMA. The illustration is adapted from a superior 3D CT–MRA fusion view of the left middle cranial fossa, emphasizing the anatomical trajectory of the MMA and its potential interface with dural drainage pathways.

Serial dynamic contrast-enhanced MRI scans were acquired from five healthy adult participants over a 6-h period, comprising one pre-contrast baseline scan (TP_baseline) and four post-contrast scans at 30, 90, 180, and 360 min (TP_30, TP_90, TP_180, TP_360, respectively) (Figure 1A; individual demographic and anthropometric characteristics for each participant are detailed in Table S1). This protocol was designed to capture the temporal dynamics of contrast accumulation and clearance across distinct anatomical compartments, with a focus on structures implicated in meningeal lymphatic and CSF-ISF drainage pathways. In murine models, the ventral meningeal region encompassing the pterygopalatine fossa and MMA has been identified as a critical site for CSF-ISF outflow.^1^^,^^3^ Consistent with these preclinical findings, our imaging strategy focused on capturing dynamic signal changes in this region to explore potential human analogues.

To quantitatively assess regional differences in contrast enhancement kinetics, we conducted a comprehensive analysis of absolute signal intensity (SI) across five anatomically defined regions (Figure 1B): the MMA internal segment (MMA-lumen), its periphery (MMA-peripheral), the nasal septum mucosa, the parasagittal dura (PSD), and the deep cervical lymph node. To standardize comparisons across individuals and anatomical compartments, all SI values were normalized to the clivus at the corresponding timepoint (Figure 1C; raw and normalized SI values across anatomical regions and timepoints shown in Table S2). This reference region was selected due to its stable anatomical position, consistent signal profile, and lack of dynamic enhancement, as independently confirmed by two board-certified neuroradiologists. This normalization strategy minimized baseline variability, enabling the robust interpretation of region-specific signal trajectories over time.

Our imaging revealed a marked 205% increase in signal intensity within the MMA-lumen at TP_30, followed by minimal signal reduction at TP_90. By TP_180, a substantial 33% decline in signal was observed, in line with the known vascular half-life (1.5–2 h) of the MR contrast agent, followed by signal plateauing at TP_360. These kinetics are indicative of a primarily vascular clearance pattern. In contrast, the deep cervical lymph node area showed a more modest <100% enhancement at TP_30, consistent with lower vascular density. Its signal decreased gradually over time but exhibited a distinct pattern between TP_90 and TP_180, with slower reduction compared to the mucosa. This delay may reflect lymphatic transit dynamics and contrast influx from central drainage sources. The PSD displayed a moderate 66% increase at TP_30, with signal decline starting at TP_90 and continuing through TP_180 and TP_360. This temporal profile suggests a hybrid vascular-lymphatic signature, lacking the rapid enhancement typical of arterial structures or the dense capillary signal seen in nasal mucosa. Instead, the PSD reflects slower flow patterns consistent with its known role in CSF-ISF drainage and lymphatic channel involvement.^8^^,^^9^^,^^10^^,^^11^^,^^12^ To complement the region-specific signal intensity findings, group-averaged, time-resolved signal enhancement curves were generated for each anatomical ROI (Figures 1D and 1E), along with corresponding plots of percentage signal increase (Figures 1F and 1G), based on data from all five participants. For each time point, signal intensity trajectories were accompanied by standard deviation (SD) values, presented in a tabular format beneath the plots to convey inter-subject variability while preserving figure clarity. These profiles revealed that the MMA-peripheral uniquely peaked at TP_90, showing sustained signal beyond vascular washout windows and resembling the trajectory of the PSD more closely than other regions—supporting the interpretation of delayed, nonvascular clearance in this ventral compartment.

### Spatiotemporal signal dynamics and quantitative regions of interest comparisons

To deepen our analysis of region-specific enhancement profiles, we systematically compared absolute signal intensity (SI) values across five anatomically defined compartments—MMA-lumen, MMA-peripheral, nasal septum, PSD, and deep cervical lymph node—at five serial timepoints (TP_0 to TP_360). These comparisons aimed to delineate distinct clearance dynamics within and beyond the dura. Corrected and normalized SI values were subjected to pairwise statistical testing between the MMA-peripheral and each comparator region to uncover kinetic patterns that might distinguish vascular from putative lymphatic behavior. The MMA-peripheral exhibited significantly higher SI than the MMA-lumen at all time points (*p* ≤ 0.005), reflecting a localized signal retention pattern. Compared to the nasal septum, MMA-peripheral showed significantly lower SI at TP_30, 90, and 180 min (*p* = 0.049, 0.034, 0.007, respectively), while TP_0- and TP_360-min values were not significantly different. MMA-peripheral SI was similar to cervical lymph node at baseline (*p* = 0.35), but significantly lower at TP_90 and TP_180 min (*p* = 0.033, 0.018). Differences at TP_30 and TP_360 min approached but did not reach statistical significance (*p* = 0.06, 0.084).

No significant differences were observed between MMA-peripheral and PSD at any timepoint (*p* > 0.54). These comparisons support the hypothesis that the MMA-peripheral exhibits distinct clearance kinetics relative to intravascular and mucosal compartments, but shares signal decay behavior with other dural regions, such as the PSD. Given that the PSD plays a key role in meningeal lymphatic drainage, this statistical similarity between MMA-peripheral and PSD strengthens our hypothesis that the MMA-peripheral may represent a critical ventral drainage interface. Collectively, these findings reinforce the interpretation that the MMA-peripheral undergoes delayed enhancement, distinct from typical vascular structures (MMA-lumen, nasal septum, cervical lymph node), and instead shares kinetic features with PSD, supporting its potential involvement in a slower clearance pathway, not attributable to arterial or venous perfusion.

Notably, the reproducibility of these signal patterns across raters and timepoints, along with the delayed enhancement profile isolated to the MMA-peripheral, strengthens confidence that these findings are not attributable to partial volume artifact. Our use of high-resolution MRI (1.0 mm isotropic), paired with inter-rater statistical agreement, supports the anatomical validity of these temporal differences. These updated analyses underscore the value of time-resolved metrics and emphasize the robustness of the MMA-peripheral signal as a non-artifactual, biologically meaningful feature.

### Distinct delayed clearance kinetics in the middle meningeal artery periphery

Among all anatomical compartments analyzed, the MMA-peripheral region exhibited a uniquely delayed enhancement profile. Unlike vascular-rich regions, which showed rapid signal peaks at TP_30 followed by washout, the MMA-peripheral demonstrated a gradual increase with peak signal intensity at TP_90, followed by a slow decline. This delayed kinetic trajectory was not observed in any other region and closely paralleled the PSD, a structure known to participate in meningeal lymphatic drainage. These findings underscore the regional specificity of delayed clearance behavior localized to the MMA periphery.

Such temporal divergence from classical vascular kinetics suggests the possibility of slower drainage mechanisms, potentially mediated by dural lymphatic or interstitial pathways. This is consistent with ventral drainage patterns reported in rodent studies and raises the hypothesis that the MMA-periphery may contribute to a human ventral outflow pathway.^1^^,^^3^ To illustrate this conceptual model, we generated a schematic diagram (Figure 1H) integrating our imaging observations with prior anatomical evidence. Importantly, we emphasize that this model synthesizes our imaging and histological findings to propose a biologically plausible framework for ventral clearance. While not intended as definitive anatomical proof, it provides a rational basis to guide future mechanistic studies.

The delayed enhancement patterns observed in the MMA-peripheral region reflect regionally distinct clearance kinetics that diverge from classical vascular behavior. While our findings do not constitute the direct functional measurement of CSF-ISF flow, the temporal signal profile strongly supports the presence of slower clearance dynamics in this compartment. These results lay the foundation for future mechanistic studies aimed at resolving the anatomical substrates and physiological pathways underlying ventral dural drainage.

### Multimodal spatial profiling of meningeal lymphatic architecture in the human dura surrounding the middle meningeal artery

**Figure 2:**
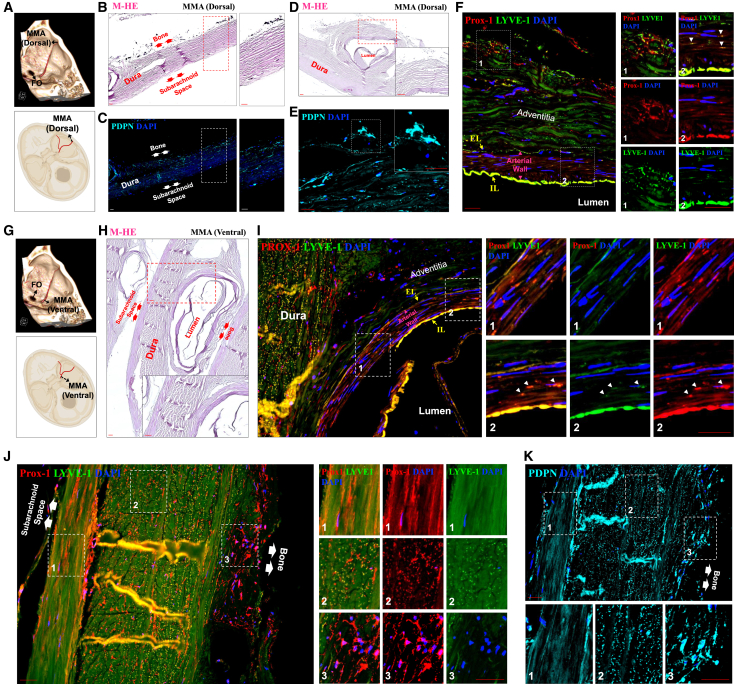
Immunofluorescence microscopy reveals lymphatic structures in the dorsal and ventral segments of the human MMA (A and G) A fused 3-dimensional reconstruction of computed tomography and magnetic resonance angiography illustrating the MMA on the calvarial surface, with the schematic demarcation of biopsy regions. Sampling sites included the dorsal (A) and ventral (G) segments of the MMA. (B, D, and H) The dissected (B and D) dorsal and (H) ventral segments were sectioned axially and counterstained with M-HE, shown in purple, to correlate their respective immunofluorescence images. The double arrows in the M-HE counterstaining indicate the positioning of the lateral dural layers relative to the subarachnoid space and bone. The inset images provide high-resolution, magnified views of the areas marked with white dashed lines. (C–F and I–K) The dural layers surrounding the (C–F) dorsal and (I–K) ventral segments of the MMA were co-immunostained for Prox-1 (red) and LYVE-1 (green) and single-stained for PDPN (cyan) to confirm their lymphatic identity. In both (B–C) (dorsal segment of MMA) and (J–K) (ventral segment of MMA) images, paired arrows denote the bone and subarachnoid space, clarifying anatomical orientation. The white arrowheads mark lymphatic signals within the arterial walls, where both markers are colocalized. The yellow arrows indicate autofluorescence associated with the internal (IL) and external (EL) elastic laminae. Nuclei were counterstained with DAPI (blue) for visualization. Inset images provide high-resolution magnified perspectives of specific areas, delineated by white dashed boxes and numbered for reference within the related immunofluorescence images. Scale: 50 μm.

To anatomically validate the CSF-ISF drainage patterns observed by MRI, we performed detailed histological mapping of the dural compartments adjacent to both dorsal and ventral segments of the MMA in the human brain. Axial sections were obtained from a neurologically intact individual, spanning the dura overlying the subarachnoid and osseous surfaces. We defined the ventral MMA as the 4 cm segment extending from the foramen spinosum (Figure 2G), with more rostral portions categorized as the dorsal MMA (Figure 2A).

**Figure 3:**
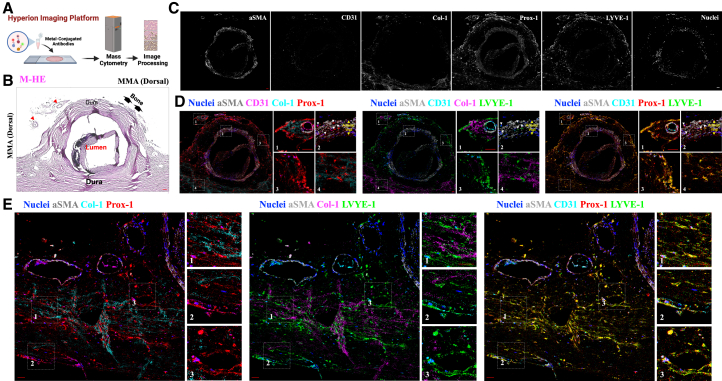
Imaging mass cytometry reveals lymphatic structures in the dorsal segment of the human MMA (A) Schematic representation of the Hyperion IMC platform. (B) The dissected dorsal MMA was cut into axial sections and counterstained with M-HE. Red arrowheads denote small meningeal arteries surrounding the MMA. (C) Representative IMC images showing marker distribution and expression profiles in the dorsal MMA region. Signals are shown for αSMA (gray), CD31 (cyan), Col-1, Prox-1 (red), LYVE-1 (green), and nuclear staining (blue). (D and E) Representative images illustrate the signaling contributions of specific markers: αSMA (gray), CD31 (cyan), Prox-1 (red), LYVE-1 (green), and nuclear staining (blue), (D) localized in the adventitia of the dorsal MMA walls, and (E) in the adjacent dural layers surrounding the dorsal segment of the MMA. The non-overlapping expression profiles between Col-1 and the lymphatic markers Prox-1 and LYVE-1 further distinguish extracellular matrix components from lymphatic elements. The co-expression of Prox-1 and LYVE-1 underscores a pronounced lymphatic identity in the (E) dural layers adjacent to the dorsal segments of the MMA and (D) its arterial wall. White arrowheads indicate the presence of lymphatic signals within the arterial walls where the colocalization of both Prox-1 and LYVE-1 occurs. Inset images provide high-resolution, magnified views of specific areas, outlined by white dashed boxes and numbered for reference in the related image. Scale: 50 μm.

**Figure 4:**
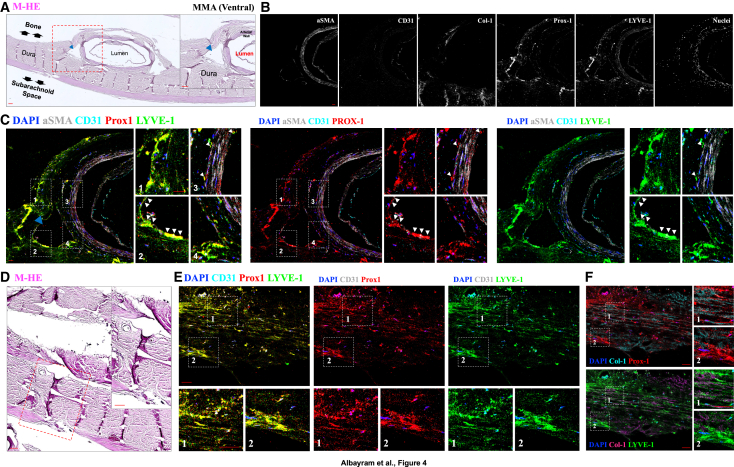
Imaging mass cytometry reveals lymphatic structures in the ventral segment of the human MMA (A) M-HE counterstaining of axial sections from the dissected ventral segment of the MMA. Blue arrowheads denote small meningeal veins adjacent to the MMA. Double arrows indicate the anatomical relationship between the dura, subarachnoid space, and underlying calvarial bone. Inset box (red dashed line) highlights the region magnified in subsequent panels. (B) Marker-specific IMC images showing expression profiles of αSMA (gray), CD31 (cyan), Col-1, Prox-1 (red), LYVE-1 (green), and nuclear staining (blue) in the ventral MMA region. (C) Representative images demonstrate the co-expression of Prox-1 (red) and LYVE-1 (green) effectively confirm the strong lymphatic identity within the dural layers surrounding the ventral segments of the MMA, as well as the adjacent dural veins and arterial walls. The white arrowheads indicate lymphatic signals in the adjacent dural veins and the arterial walls of MMA, where both markers are colocalized. (D) The M-HE counterstaining and the inset image delineated by red dashed lines highlight the area pertinent to the subsequent IMC analysis. (E) The co-localization of Prox-1 (red) and LYVE-1 (green) provides clear evidence for a distinct and well-established lymphatic pathway within the dural layers near the ventral segments of the MMA. (F) The non-overlapping co-expression profiles of Col-1 in relation to the lymphatic markers Prox-1 and LYVE-1. Inset images offer high-resolution magnified perspectives of specific areas, delineated by white dashed boxes and numbered for reference within the related image. Scale: 50 μm.

To overcome the common limitations associated with single-modality analyses, we employed a dual-platform approach: high-resolution immunofluorescence confocal microscopy (Figure 2) and complementary Hyperion Imaging Mass Cytometry (IMC) (Figure 3 and 4; the list of metal-conjugated antibodies used for IMC is shown in Table 1). This orthogonal strategy enabled simultaneous spatial, molecular, and structural characterization of MLVs across multiple tissue layers. Both techniques converged on the presence of a robust and spatially organized lymphatic network, with marked enrichment in the ventral dura, particularly near the axial level of the pterion.

**Table 1:** Hyperion imaging mass cytometry antibody panel for human dural tissue analysis

Metal	Antibody	Vendor	Catalog #	Clone	Lot ID	Dilution
113In	total H3	CST - Cell Signaling Technologies	4499	D1H2	17	1:400
141Pr	aSMA	Fluidigm	3141017D	1A4	2212501-22	1:300
151Eu	CD31	abcam	ab207090	EPR3094	1028609-3	1:50
154Sm	Prox1	Novus Biologicals	NBP1-30045	5G10	B-14	1:25
158Gd	PDPN	Novus Biologicals	NBP2-54347	PDPN/1433	10630-1PABX221020	1:50
160Gd	Lyve1	R&D Systems	AF2089	polyclonal	KPY0122121	1:25
169Tm	Col_A_1	Fluidigm	3169023-D	polyclonal	2111779-09	1:400

List of metal-conjugated antibodies used for Imaging Mass Cytometry (IMC) to profile lymphatic, vascular, and structural markers in human dural tissue surrounding the MMA. Each entry includes target antigen, clone, metal tag, vendor, catalog number, and dilution. All antibodies were applied in a single-step cocktail and validated on control tissues. Panel design enabled multiplexed spatial analysis of lymphatic architecture and supporting structures.

### Lymphatic topography and structural features differ between dorsal and ventral middle meningeal artery regions

In the ventral segment of the periosteal dura, we identified the robust expression of canonical lymphatic markers—PROX1, PDPN, and LYVE1—within the periosteal dura, particularly in the ventral segment, where signal intensity increased progressively toward the bony interface (Figures 2B–2C, 2J–2K, 3B–3D, and 4A–4C). This distribution aligns with recent reports of skull bone marrow–dura connectivity in myeloid trafficking that facilitates myeloid trafficking into the CNS following injury. Although lymphatic CSF–bone outflow has been demonstrated in rodent models,^13^ our findings offer a spatial validation of this anatomical interface in the human MMA.

Although no lymphatic signal was detected within the adventitial layer of the MMA arterial wall, we observed interspersed PROX1+, PDPN+, LYVE1+ cells within the tunica media in both dorsal and ventral segments (Figures 2F and 2I). To distinguish signal from elastic lamina autofluorescence—commonly misinterpreted as lymphatic structures in fluorescence microscopy—we cross-validated these features using IMC, which is free from autofluorescent interference (Figures 3D and 4C). This finding demonstrates the anatomical presence of lymphatic marker-positive cells within the arterial media of the MMA. While these cells localize to a region of potential fluid or cell transport, their functional significance remains unknown. If these structures support fluid movement along arterial walls, they may parallel the intramural periarterial drainage pathways (IPAD) proposed in the context of glymphatic-lymphatic exchange.^14^^,^^15^^,^^16^ These observations highlight the structural complexity of the MMA region, including previously unreported lymphatic associations with both the periosteum and arterial wall. However, we emphasize that these are anatomical findings, and further studies will be needed to determine whether these lymphatic elements play an active role in drainage or homeostasis.

Notably, the ventral MMA dura displayed distinct orientation patterns of lymphatic vessels across dural layers: inner dura showed anterior–posterior alignment, while middle layers followed a superior–inferior axis, and outer dura revealed a complex, multidirectional lymphatic pattern (Figures 2J, 2K, 3E, and 4E–4F). These regional differences were most prominent in axial sections of the ventral MMA, where we identified elongated PROX1-, PDPN-, and LYVE1-positive structures situated between the inner and middle dural layers. Although their morphology exhibits a tubular appearance suggestive of vessel-like organization, their precise functional identity remains undefined. At present, these observations should be interpreted as anatomical findings. Definitive insight into their physiological role, whether related to lymphatic drainage, immune trafficking, or structural support, will require future studies employing tracer-based methods or *in vivo* functional imaging.

### Cross-validation of lymphatic identity and exclusion of autofluorescent artifacts

To ensure the specificity of our findings and eliminate potential false positives, we employed a rigorous validation strategy across both confocal immunofluorescence microscopy and Hyperion IMC.^17^^,^^18^^,^^19^^,^^20^^,^^21^ Negative and positive tissue controls, including human tonsil, prostate, and colorectal sections, were processed identically (Figures S2 and S3). Notably, lymphatic markers did not colocalize with type I collagen, the principal structural component of the dura mater (Figures 3D, 3E, and 4F), supporting the anatomical distinction between MLVs and fibrous dural elements.^22^^,^^23^^,^^24^

Despite the single-brain origin of this histological validation study, the anatomical and molecular features we observed were consistently detected across two orthogonal high-resolution imaging platforms, substantially reinforcing the robustness of our findings. Crucially, this histological analysis was designed to complement and validate the dynamic contrast-enhanced MRI results obtained from five healthy human participants, serving as spatial and cellular confirmation of the CSF-ISF drainage patterns and regional signal behaviors identified *in vivo*. By integrating *in vivo* functional imaging with *ex vivo* anatomical validation, we provide a convergent framework for identifying regionally distinct lymphatic features in the human dura, particularly surrounding the middle meningeal artery.

Moreover, this study represents an application of Hyperion IMC^25^^,^^26^^,^^27^ to human meningeal tissue for the spatial profiling of lymphatic structures, and to our knowledge, the use of this platform to interrogate anti-lymphatic features within the human CNS. This technical innovation, together with the high-resolution validation from confocal microscopy, establishes a methodological foundation for future efforts to anatomically resolve the meningeal lymphatic system in health and disease.

Together, these findings support a complex and spatially stratified meningeal lymphatic system in the human brain. Our data, validated across two complementary modalities and revealed in human tissue using IMC, underscore the feasibility and value of high-resolution spatial proteomics in mapping CNS drainage pathways.

## Discussion

Animal studies have established the ventral meningeal region—particularly around the MMA and pterygopalatine fossa—as a critical site for CSF and ISF drainage via MLVs.^1^^,^^3^ However, whether similar drainage pathways operate in the human brain, especially within ventral compartments, has remained unclear. While one recent case report^5^ noted CSF/ISF accumulation around the MMA in a patient with Gorham-Stout disease, its pathological context limits generalization to healthy physiology.

In this study, we used dynamic contrast-enhanced MRI in five healthy adults to investigate CSF-ISF transport around the MMA and uncovered a distinct, previously undescribed drainage signature. Specifically, signal intensity in the MMA periphery peaked at 90 min post-injection—substantially delayed compared to other anatomical compartments such as the arterial lumen, PSD, nasal mucosa, and cervical lymph nodes, all of which peaked at 30 min. The delayed and attenuated enhancement around the MMA did not follow typical vascular kinetics, suggesting the presence of a slower, possibly lymphatic flow. While these group-averaged trends and statistical comparisons strengthen the objectivity of our observations, we acknowledge the potential contribution of partial volume effects and regional anatomical variability, particularly in small ROIs. Nonetheless, the anatomical consistency of ROI placement, confirmed by high inter-rater agreement, supports the reproducibility of the observed dynamics.

Additionally, we strengthened our interpretive framework by introducing a spatiotemporal analysis subsection, as detailed in the Results. This quantitative framework incorporates temporal signal ratios and statistical comparisons across all ROIs, thereby reducing reliance on single-point peak enhancement metrics, which can be susceptible to partial volume confounds. Together, the enhanced methodology and robust inter-rater reproducibility provide further validation that the observed MMA-peripheral signal trajectory reflects a true regional clearance signature, rather than an artifact of resolution or measurement error.

While our analysis relies on visually guided ROI placement, we recognize the potential for partial volume effects to influence signal intensity measurements. To minimize this limitation, all ROIs were strategically positioned at the central portions of the target anatomical regions, avoiding boundaries that might capture adjacent tissues. This approach was independently performed by two board-certified neuroradiologists with anatomical expertise. Importantly, the robustness of ROI placement was supported by strong and statistically significant inter-rater correlations, as demonstrated by Pearson correlation analysis (Figure S3). These steps were taken to ensure the anatomical consistency and reproducibility of our spatiotemporal signal comparisons, while acknowledging the descriptive nature and scale constraints of the present dataset. Consistent with prior vascular imaging studies, the nasal mucosa and PSD regions exhibited enhancement dynamics reflecting conventional vascular wash-in and wash-out. In contrast, the MMA-peripheral showed a distinct delayed clearance profile, which we interpret as reflective of slower interstitial or lymphatic-associated drainage. This reinforces the specificity of the MMA-peripheral compartment in meningeal fluid dynamics, as opposed to the more rapid vascular kinetics observed in adjacent tissues.

The quantitative comparisons reinforce our interpretation that the MMA-peripheral exhibits a distinct spatiotemporal signature of delayed signal enhancement relative to other compared anatomical structures. Specifically, while regions such as the nasal septum and cervical lymph node demonstrated rapid signal rise and early peaks characteristic of perfusion-driven kinetics, the MMA-peripheral showed slower signal accumulation and a delayed peak near 90 min, consistent with slow clearance dynamics. Moreover, MMA-peripheral’s signal trajectory closely mirrored that of the PSD, further suggesting a dural clearance signature distinct from intravascular compartments. These findings are supported by rigorous statistical analysis of absolute signal intensity across five anatomical ROIs, with paired-sample t-tests identifying significant region- and time-dependent differences in enhancement behavior. This quantitative framework validates the temporal signal divergence observed in group-averaged curves and substantiates the interpretation of the MMA-peripheral as a potential conduit of ventral CSF–ISF clearance. The similarity in behavior between MMA-peripheral and PSD supports a shared role in the egress of dural fluid. At the same time, divergence from the nasal septum, cervical lymph node, and MMA-lumen strengthens the case for a non-arterial/venous pattern in MMA-peripheral. Together, these data provide robust and reproducible evidence that the MMA-peripheral participates in a delayed drainage compartment, consistent with slower interstitial or lymphatic-associated fluid transit.

This finding aligns with preclinical evidence and supports the presence of dural lymphatic drainage along the MMA in humans. To validate these *in vivo* findings, we performed high-resolution spatial profiling of the dura surrounding both dorsal and ventral MMA segments using two complementary histological techniques: immunofluorescence confocal microscopy and Hyperion IMC. This dual-modality approach enabled the orthogonal verification of our MRI-based observations, providing high-dimensional, marker-specific evidence of lymphatic structures. Importantly, this is an application of IMC to human meningeal lymphatic tissue, establishing a benchmark for spatial proteomic analysis in this field.

We emphasize that our dynamic MRI data provide indirect evidence of fluid transport pathways in the ventral dura, inferred from signal enhancement kinetics rather than direct tracer tracking or flow measurements. The delayed enhancement around the MMA-peripheral is consistent with slower clearance dynamics, which may reflect lymphatic involvement; however, alternative explanations, such as interstitial diffusion or perivascular retention, cannot be excluded. Our interpretation aligns with prior rodent studies that describe ventral lymphatic outflow zones; however, definitive confirmation in humans requires future mechanistic investigation.

Our histological results confirmed the presence of a dense and structurally distinct lymphatic network along the MMA, particularly enriched in ventral segments near the foramen spinosum. PROX1, PDPN, and LYVE1 markers delineated multilayered lymphatic architecture across the inner, middle, and outer dural layers, with tubular, anterior–posterior lymphatic alignment in the inner dura, a superior–inferior orientation in the middle dura, and more complex patterns in the outer dura. These distinct orientations may reflect regional adaptations in fluid clearance capacity or connectivity with extracranial structures. The identification of elongated, lymphatic marker-positive structures within the ventral dura constitutes an anatomical observation within the human meningeal lymphatic landscape. While their vessel-like morphology and expression of PROX1, PDPN, and LYVE1 are consistent with a lymphatic identity and suggest an organized architectural arrangement, our data do not establish their role in active drainage. These findings should be interpreted as anatomically grounded observations that expand upon rodent studies of ventral meningeal lymphatics, while underscoring the need for future functional validation in human tissue.

We also observed PDPN+ and PROX1+ signal—without LYVE1 colocalization—within the periosteal dura and adjacent to bone, supporting a mesenchymal-like lymphatic phenotype and hinting at immunologic or structural crosstalk with the skull bone marrow. These findings parallel recent reports describing direct connections between the meninges and calvarial bone marrow that mediate immune cell trafficking and fluid exchange.^13^ Of particular note, lymphatic markers were detected within the tunica media of the MMA, raising intriguing possibilities about periarterial lymphatic drainage mechanisms akin to the IPAD system described in murine models.^14^^,^^15^^,^^16^ IMC-based absence of autofluorescence artifacts in this region strengthens confidence in this observation, which may suggest hybrid vascular-lymphatic functionality within meningeal arteries. The detection of robust lymphatic marker-positive elements within the arterial media and periosteal surfaces adds an anatomical dimension to our understanding of ventral dural lymphatics. While this finding parallels rodent studies describing lymphatic-perivascular interactions, we emphasize that our data reflect marker localization alone and do not establish a functional role in perivascular drainage or vessel remodeling. It remains possible that these lymphatic elements contribute to tissue homeostasis or immune surveillance rather than fluid clearance. Future studies employing live imaging or molecular manipulation will be needed to clarify these roles.

While our findings align anatomically with rodent models describing IPAD, we emphasize that our data in human tissue are descriptive and do not provide functional evidence of drainage activity. The presence of lymphatic markers in the arterial media, while intriguing, does not confirm involvement in clearance pathways or vessel remodeling. These possibilities remain hypotheses that warrant testing in future human or translational studies.

A striking molecular feature emerged from our dual-platform analysis: PROX1 and PDPN showed strong, consistent expression, whereas LYVE1 was faint and discontinuous, particularly in ventral regions. This divergence likely reflects functional and developmental heterogeneity within human MLVs. While PROX1 and PDPN are well-established markers of lymphatic endothelial identity, with PDPN also linked to mesenchymal-like properties, LYVE1 is more closely associated with classical lymphatic endothelium and is often downregulated in pre-collecting or specialized lymphatic structures. Our findings align with previous research showing that some lymphatic vessels, especially those in specialized vascular environments, can express PROX1 and PDPN even in the absence of LYVE1. This pattern, also described in so-called hybrid vessels, reflects the broader molecular and functional diversity within the lymphatic system.^28^^,^^29^^,^^30^ These findings highlight the necessity of using multi-marker strategies and orthogonal validation methods for accurate lymphatic identification in complex tissues such as the human dura. Our histological analyses revealed divergent expression patterns of PROX1, PDPN, and LYVE1 across the dural layers of the human MMA region. While PROX1 and PDPN were consistently detected in both inner and outer dural compartments, LYVE1 expression was more limited, particularly in ventral segments. These patterns suggest molecular heterogeneity within human meningeal lymphatics, which may reflect functional stratification analogous to that described in peripheral lymphatic vessels.^28^^,^^31^ However, we emphasize that this interpretation remains hypothetical and will require further studies to establish whether these molecular differences translate to distinct physiological roles in lymphatic drainage or immune modulation.

We acknowledge several limitations. First, the MRI cohort consisted of only five participants and was limited to five time points over a 6-h period. A longer or more frequent imaging protocol might reveal additional flow dynamics, though clinical feasibility remains a constraint. Additionally, the small size and variability of the MMA limited us to manual measurements; despite attempts to use two automatic or semi-automatic techniques, all quantification relied on double-blind manual assessments by two neuroradiologists to ensure data accuracy. Second, histological validation was performed on a single postmortem specimen. However, we mitigated this by using two highly complementary imaging platforms (confocal and IMC) and integrating our findings with *in vivo* MRI results from multiple individuals. Although the use of freshly dissected specimens would be ideal, such access remains limited. Fixation with formalin and ethanol can sometimes alter antigenicity by changing protein structure or masking key epitopes. Imaging mass cytometry also presents certain technical constraints, such as the need to apply all antibodies at once and limit fixation to a single step—factors that may affect the signal quality of some markers. Even so, the use of metal-tagged antibodies in IMC avoids spectral overlap and eliminates autofluorescence, making it a powerful complementary tool for validating lymphatic structures.

Lastly, marker specificity poses interpretive challenges. PDPN, although a widely accepted lymphatic marker, is also expressed in the perineurium, mesothelial cells, and basal epidermal layers.^30^^,^^32^^,^^33^ LYVE1, similarly, is found in alternatively activated macrophages and other non-lymphatic populations.^34^^,^^35^ Prior studies have documented variable or undetectable expression of these markers in different tissues and conditions.^36^^,^^37^^,^^38^^,^^39^^,^^40^^,^^41^^,^^42^ This biological variability necessitates cautious interpretation.

Together, our findings provide multimodal, *in vivo*, and *ex vivo* evidence of a structurally distinct meningeal lymphatic network surrounding the human MMA. This region, well-established in rodents as a ventral lymphatic outflow hub, may similarly serve as a functional CSF-ISF drainage route in humans. Our work adds a critical dimension to the anatomical map of human CNS lymphatic pathways and sets the stage for future investigations into how these pathways contribute to fluid clearance, immune surveillance, and neurological disease vulnerability. Taken together, our *in vivo* and *ex vivo* findings provide complementary insights into the anatomical landscape and drainage dynamics of the ventral MMA region. However, we emphasize that the observed spatial co-localization does not constitute direct evidence of functional coupling between delayed MRI enhancement and lymphatic drainage. Further studies employing real-time tracer tracking or combined imaging-histological validation will be required to establish whether these anatomical lymphatic structures actively mediate ventral drainage in humans.

### Limitations of the study

This work is anatomical and observational by design. The dynamic contrast–enhanced MRI cohort was small (*n* = 5) and sampled at five time points over ∼6 h, which constrains temporal resolution and generalizability across age, sex, and comorbidity. ROI definition relied on visually guided placement around small ventral structures; although two board-certified neuroradiologists performed double-blind measurements with strong inter-rater agreement, and we supplemented peak metrics with a spatiotemporal analysis framework, partial-volume effects and regional variability may still influence signal trajectories. The delayed enhancement in the MMA-peripheral compartment is therefore consistent with slower interstitial or lymphatic-associated transit but does not constitute direct evidence of lymphatic flow; alternative explanations (e.g., interstitial diffusion or perivascular retention) cannot be excluded. *Ex vivo* validation derives from a single human dura specimen. Fixation and tissue handling can alter antigenicity, and imaging mass cytometry, while reducing spectral overlap and autofluorescence, requires single-step antibody cocktails that may differentially affect marker detection. Marker specificity also limits inference: PROX1/PDPN/LYVE1 patterns suggest a lymphatic-like identity, but these proteins can also appear in non-lymphatic cell types, and faint or discontinuous LYVE1 expression emphasizes molecular heterogeneity. The detection of lymphatic markers within the arterial tunica media is descriptive and should not be taken as functional proof of periarterial drainage. Larger, demographically diverse cohorts with higher temporal sampling, automated segmentation, live tracer tracking, and additional fresh human tissue, ideally integrating orthogonal molecular assays, will be needed to test mechanism and establish ventral drainage function in humans.

## Resource availability

### Lead contact

Requests for further information and resources should be directed to and will be fulfilled by the lead contact, Onder Albayram, Ph.D. (albayram@musc.edu).

### Materials availability

This study did not generate new, unique reagents.

### Data and code availability

Data: No standardized dataset was generated in this study. All raw data, experimental and analysis methods, and original images supporting the findings of this study are available upon request from the corresponding author.

Code: This study does not report original code.

Additional information: Any additional information required to reanalyze the data reported in this article is available from the lead contact upon request.

## Acknowledgments

The authors gratefully acknowledge Hammerbacher family for their generous donation of the imaging system to our laboratory. This work was supported by research grants from the South Carolina Alzheimer’s Disease Research Center (10.13039/100006528ADRC) Pilot Grant and the COBRE in Neurodevelopment and Its Disorders P20GM148302 Research Grant to O.A.; the 10.13039/100000104National Aeronautics and Space Administration (NASA) directed study and the 10.13039/100000006Office of Naval Research (N0014-20-1-2463) to R.D.S., and S.B.R.; VA Merit Review (BX000347), VA Senior Research Career Scientist Award (IK6 BX004471), 10.13039/100000002NIH RF1 NS083559, and RF1 NS10457 to A.E.

## Author contributions

M.A. and O.A. designed the study. M.A., O.A., S.B.R., R.D.S., and K.Y. coordinated and directed the project. M.A., S.B.R., I.S.T., S.B.R. collected clinical and imaging data. K.Y., M.M., E.L., and Y.S.K. dissected the human medial meningeal artery from the human brain. E.K., A.E., and O.A. collected immunofluorescence microscopy data. H.R. and O.A. collected imaging mass cytometry data. M.A., S.B.R., F.T., H.R., and O.A. wrote the report. M.A., S.B.R., F.T., F.B., K.Y., R.D.S., and O.A. provided scientific direction. M.A., S.B.R., I.S.T., and O.A. performed image analysis. M.A., S.B.R., F.T., and O.A. performed data analysis. F.T. performed statistical analysis.

## Declaration of interests

All the authors declare no competing interests. Declaration of generative AI and AI-assisted technologies in the writing process. All authors declare that AI and AI-assisted technologies were not used in the preparation of this article.

## STAR★Methods

### Key resources table

**Table:** 

REAGENT or RESOURCE	SOURCE	IDENTIFIER
**Antibodies**		

Prox-1 (5G10)	Novus	NBP1-30045PE; RRID:AB_3205294
LYVE-1	Millipore	AB2988; RRID:AB_10615605
PDPN (1443)	enQuire BioReagents	10630-MSM1-P1
Alexa Fluor 488	Invitrogen	A11008; RRID:AB_143165
Alexa Fluor 594	Invitrogen	A11032; RRID:AB_2534091
Total H3 (D1H2)	Cell Signaling	4499; RRID:AB_10544537
aSMA (1A4)	Fluidigm	3141017D; RRID:AB_2890139
CD31 (EPR3094)	Abcam	ab207090; RRID:AB_2889382
PDPN (1433)	Novus	NBP2-54347; RRID:AB_333367
Lyve-1	R&D Systems	AF2089; RRID:AB_355144
ColA1	Fluidigm	3169023-D; RRID:AB_2810857

**Chemicals, peptides, and recombinant proteins**		

Gadobutrol	Gadovist, Bayer	NDC-50419-325
Formalin	Fisher Chemical	SF98-4
Histo-Clear II	Electron Microscopy Sciences	64111-01
TE Buffer, Tris-EDTA	Fisher BioReagents	BP2473500
Goat serum	Invitrogen	50062Z
Phosphate-buffered saline	Thermo Scientific Chemicals	J62036K2
Mayer’s (Lillie’s Modification)	Volu-Sol™	VMH032
Eosin Y	Fisher Chemical	SE23-500D
CitriSolv™ Hybrid Solvent	Decon	04-355-121
Triton X-100	MilliporeSigma	MTX15681

**Software and algorithms**		

SPSS v20.0	IBM	20.0

### Experimental model and study participant details

#### Human participants

This study was approved by the Institutional Review Board (IRB) of the University of Florida (Gainesville, Florida, USA) under protocol #IRB201902542, titled *“Glymphatic Function in Extreme Environments.”* Written informed consent was obtained from all participants in accordance with the Declaration of Helsinki. A total of five healthy adult volunteers were included in the final imaging analysis. Individual demographic and anthropometric data, including age, sex, height, weight, body mass index (BMI), race, and education level, are provided in Table S1. All enrolled participants were screened and confirmed to be free of hypersensitivity to contrast agents, severe allergic conditions, or renal dysfunction (defined as glomerular filtration rate <30 mL/min/1.73 m^2^), consistent with safety guidelines for gadobutrol administration. Additionally, all participants were free of any (*self-reported*) medical or neurological health conditions and were compliant with medical safety standards for MRI, which includes being free of any implanted medical device, metallic foreign bodies, or were pregnant and/or breastfeeding. Data were captured from five participants (3 males | 2 females, 29 ± 12 years, height 1.73 ± 0.14 m, weight 74.3 ± 25.1 kg, body mass index 24.4 ± 5.4 kg m^2^ [males: 33 ± 15 years, 1.72 ± 0.14 m, 74.8 ± 26.1 kg, 25.1 ± 6.3 kg m^2^] [females: 23.5 ± 0.7 years, 1.74 ± 0.20 m, 73.5 ± 34.0 kg, 23.4 ± 5.9 kg m^2^]) that were free of any (*self-reported*) medical or neurological health conditions. Participants were recruited using flyers in and around the institution.

### Method details

#### MR methodology

##### Experimental procedures

We previously identified robust CSF-ISF drainage and possible lymphatic flow surrounding the sinuses using 3D T2-weighted Fluid Attenuated Inversion Recovery (FLAIR) magnetic resonance imaging,^8^ specifically focusing on the dura surrounding the MMA lymphatic pathways (Figure S4). All participants underwent five MR scans. Imaging commenced at 8 AM with identical MRI scans performed across TP and participants, with a scan duration of 35 min. An intravenous injection of 0.1 mL or 1 mmol/mL gadobutrol (Gadovist, Bayer) per kilogram of body mass was performed immediately following TP_baseline_ image acquisition to enhance the visualization of CSF pathology. The attending neuroradiologist (M.A.) monitored participants for adverse reactions to the contrast. Throughout all study procedures, participants were instructed to stay awake and lie on their backs while positioned in the MRI during image acquisitions and on a stretcher outside the MR control room between MRI acquisitions. Participants were advised to remain in the supine position except during restroom breaks, mealtimes, or when being transported, such as when transitioning to the MR scanner. Durations spent in the upright position were recorded for each participant. Further details about the policies and procedures of the experimental design can be found in our recent publication.^43^

##### Neuroimaging

Imaging was performed at UF’s McKnight Brain Institute on a Philips 3T Ingenia Elition X MR-scanner (Koninklijke Philips N.V.) with a 32-channel head coil. We acquired several different MRI sequences; here, we report analyses on the optimized T1 CS-BB sequence with motion-sensitization driven equilibrium (MSDE) [acquisition time: 2 min and 17 s]. The pulse sequence parameters were established at a TR/TE = 700/35 ms, flip angle = 80°, FOV = 200 × 240 × 160, acquisition matrix = 200 x 240, slices = 321, orientation = sagittal, slice gap = −0.5 mm, fast imaging mode = TSE (55, es/shot = 5.0/305 ms), and a spatial resolution of 1.0 mm isotropic voxel. During all imaging procedures, participants focused on a white cross displayed against a black background to minimize eye movements in the scanner.

##### Neuroimaging analysis

T1 CS-BB images were assessed by two separate board-certified neuroradiologists (M.A. and I.S.T.) with a combined 40 years of experience. The neuroradiologists were double-blinded to participant demographic information (i.e., age, sex, etc.). Due to the small size and high variation in the neuroanatomy of the evaluated structures, the neuroanatomical regions of interest (ROIs) were manually drawn by each neuroradiologist separately. Circular 0.4 mm^2^ ROIs (diameter of 0.7 mm) were drawn in the MMA (lumen and peripheral), nasal septum, cervical lymph node, parasagittal dura (PSD), and clivus to evaluate signal changes. As illustrated in Figure 1B, MMA (lumen) ROI is shown in maroon and overlaid on the participants' T1 CS-BB image in native space (Figure 1A). MMA (peripheral) ROI is shown in bright green (Figure 1B). Clivus matter ROI is shown in pink (Figure 1C). Nasal septum mucosa is shown in orange (Figure 1D). The lymph node area ROI is dark green (Figure 1E), corresponding to the anatomical region where cervical lymph nodes are typically located according to standard anatomical references. The PSD ROI is blue (Figure 1F). Each anatomical structure was measured in the projections that depicted the maximum signal intensity. All ROI signal intensities were normalized to the signal of the clivus at the corresponding timepoint to control for inter-subject variability and enhance comparability across anatomical regions. Neuroradiologist measurements began with the MMA (lumen and peripheral), orientated by the most prominent (i.e., largest and brightest) segment of the ROI and providing laterality of subsequent ROIs, i.e., if the most prominent was the right-side, then all remaining ROIs were ipsilateral to the initial measure, across time points. In the dataset, four of the five participants displayed right-side dominance and had acquisitions from the right side. Placement and signal intensity extractions from all six ROIs, across participants and TP, were executed by both neuroradiologists (Figure 3S) using the Picture Archiving and Communication Systems (PACS/Visage 7.1.14, Visage Imaging).

To further mitigate the potential influence of partial volume effects—especially critical given the small ROI size and regional anatomical heterogeneity—we conducted a quantitative analysis that expanded upon our original methodology. This updated approach incorporated time-normalized signal ratios (TP_30/0, 90/0, and 90/30) across anatomically defined ROIs and was performed using corrected signal intensities extracted independently by two neuroradiologists. These inter-rater analyses confirmed strong reproducibility and anatomical consistency, while our fine-scale voxel size (1.0 mm^3^) minimized artifact risks. The addition of this spatiotemporal analysis provides enhanced methodological granularity and bolsters interpretability of region-specific clearance dynamics.

#### Dissection of a human brain specimen in dorsal and ventral segments of MMA

A formalin-fixed adult human cadaver head, devoid of intracranial or extracranial pathologies, was employed for dissection purposes (Male, Age: 59, Weight: 135 lbs). The dissection was performed using microsurgical instruments under X6 to ×40 magnifications provided by a Zeiss Surgical Microscope (Carl Zeiss AG, Oberkochen, Germany). After the coronal scalp incision, the calvarium was removed without dura injury, and MMA with surrounding dura (2 cm off from the artery on each side) was harvested from the same axial level of the pterion to the level of the foramen spinosum. The ventral MMA is defined as the segment extending 4 cm from the foramen spinosum, whereas the portion beyond this 4 cm mark is classified as the dorsal MMA. The specimen was stored in a 5% formalin solution. Approval for brain donation was secured from the Institutional Review Board at the University of Virginia, and our studies involving human samples have been sanctioned by the Institutional Review Boards at both the University of Virginia and the Medical University of South Carolina.

##### Immunofluorescence microscopy: Immunohistochemistry

Following the dissection of formalin-fixed dorsal and ventral segments of the MMA, the specimens were embedded in paraffin wax, as outlined in our previous publication.^44^^,^^45^^,^^46^^,^^47^^,^^48^^,^^49^ Subsequently, Formalin-Fixed Paraffin-Embedded (FFPE) tissue blocks were sectioned to a thickness of 5 microns using a rotary microtome (Leica, Germany) and placed on charged microscope slides. The FFPE sections underwent deparaffinization [Histoclear II (2 × 5 min each)] and rehydration using a series of ethanol washes as follows: 100% Ethanol (2 × 5 min each), 90% Ethanol (5 min), 70% Ethanol (5 min), ddH2O (5 min). Following deparaffinization and rehydration, the tissue slides underwent a brief boiling step in Tris-EDTA buffer to facilitate antigen retrieval. Primary antibodies [Prox-1 (5G10), Novus, NBP1-30045, diluted 1/250], [LYVE-1, Millipore AB2988, diluted 1/250], and PDPN (1443), enQuire BioReagents, 10630-MSM1-P1, diluted 1/400) were applied and allowed to incubate overnight at 4°C. For double immunofluorescence, the sections were incubated with isotype-specific secondary antibodies conjugated to Alexa Fluor 488 or 594 (Invitrogen A11008 and A11032) for 1 h at room temperature. Goat serum (50062Z, Invitrogen) was the blocking agent throughout the staining process. After each incubation step, the sections were thoroughly washed four times with PBS. The labeled sections were visualized using a Zeiss confocal microscope or a Keyence imaging system (X800).

##### Mayer’s hematoxylin and eosin (M-HE) counterstaining

The tissue blocks were sectioned at 5 microns and deparaffinized before being affixed to positively charged microscope slides. Then, histological staining was conducted using Mayer’s hematoxylin (Lillie’s Modification; Dako North America, Carpinteria, CA) and eosin Y solution (Thermo Fisher Scientific, Waltham, MA).

##### Hyperion imaging platform: Tissue deparaffinization and antigen retrieval

pH9 Tris-ETDA buffer antigen retrieval solution was prepared and aliquoted in 50 mL Falcon tubes at a volume of 40 mL each and were preheated in a water-bath set to 95°C. The slides were deparaffinized in 100% CitriSolv, 2X for 15 min each with gentle agitation every 3 min, then rehydrated with a series of ethanol solutions: 2X in 100%, 1X in 80%, 1X in 70%, all for 5 min each, with gentle agitation every 2 min. After the 70% ethanol, the slides were washed in MilliQ water for 5 min, with gentle agitation every 2 min. Two slides were placed in a back-to-back configuration in each 95°C-preheated tube to incubate while rotating in the HL-2000 HybriLinker hybridization oven for 30 min. As the tissue was sensitive, the HybriLinker was the preferred method to a water bath for this antigen retrieval step to ensure the maintenance of the 95°C temperature throughout the designated time. After removal from the oven, the slides cooled at room temperature for 15 min. After cooling, the slides were removed from the antigen retrieval solution and washed in 1X TBS on a shaker 3X for 10 min each.

##### Tissue staining

The slides were placed into a hydration chamber and gently dried around the tissue with KimWipes. Each tissue core was carefully outlined using a PAP hydrophobic pen. SuperBlock (PBS) Blocking Buffer was then applied to the tissue areas, and the slides were incubated in the hydration chamber at room temperature for 1 h. Before starting the experiments, control tests on various antibody dilutions were conducted to refine the antibody panel and protocol below (Table 1).

The antibody cocktail was prepared using the appropriate dilutions of conjugated antibodies, 1X PBS, and 10% BSA. Following the removal of the blocking buffer, the antibody mixture was applied to the tissue sections and incubated overnight at 4°C in the hydration chamber. The following day, the slides were washed twice for 10 min each with 0.2% Triton X- in 1X PBS while agitating on a shaker, followed by two 10-min washes in 1X TBS. The tissues were then stained with an iridium-intercalator solution diluted 1:400 in 1X PBS and incubated for 30 min at room temperature in the hydration chamber. After staining, the slides were washed for 5 min in 1X TBS, followed by another 5-min wash in MilliQ water. The slides were left to air dry at room temperature for at least 20 min before undergoing Hyperion acquisition.

##### Hyperion image acquisition

After the tuning and quality control were completed, a single image was captured per slide using Hyperion. Panoramic images were generated to include the tissue areas that the CyTOF software could read, and ROI was selected based on the M-HE-stained images of the tissues. The antibody panel, with its specific conjugated metals, was imported and designated for each ROI before beginning the ablation and data acquisition process.

### Quantification and statistical analysis

#### MRI acquisition and statistical analysis

Dynamic contrast-enhanced T1-weighted MRI scans were performed in five healthy adults across five timepoints: baseline (TP_0) and post-intrathecal contrast at 30, 90, 180, and 360 min (TP_30–TP_360). Imaging protocols were maintained constant across time points to ensure comparability. Five anatomically defined regions of interest (ROIs) were selected: MMA lumen (MMA-lumen), MMA periphery (MMA-peripheral), nasal septum mucosa, parasagittal dura (PSD), and deep cervical lymph node. ROIs were manually placed by two board-certified neuroradiologists based on anatomical landmarks, and carefully positioned within the center of each compartment to minimize partial volume contamination. Inter-rater agreement was confirmed using Pearson correlation analysis (see Figure S3). For each ROI and timepoint, signal intensity (SI) values were background-corrected and averaged across participants. Group-level SI trajectories were visualized using line plots with standard deviation (SD) values included in tabular overlays to convey inter-subject variability. To quantify regional differences in enhancement kinetics, we performed paired-sample t-tests comparing absolute SI values between MMA-peripheral and each comparator region (MMA-lumen, nasal septum, PSD, lymph node) at each timepoint. All statistical analyses were performed using SPSS v20.0 (IBM, Armonk, NY). Continuous variables were expressed as mean ± SD, and categorical variables as frequencies and percentages. Inter-regional comparisons were conducted using two-tailed paired samples t-tests. Correlations between radiologist assessments were evaluated using Pearson correlation tests. Statistical significance was set at *p* < 0.05.
