# Supplementary material for: Meningeal lymphatic architecture and drainage dynamics surrounding the human middle meningeal artery
Source: iScience. 2025 Oct 4;28(11):113693. doi: 10.1016/j.isci.2025.113693 (PMC12552907; doi:10.1016/j.isci.2025.113693)
Supplement: Document S1. Figures S1–S4 and Tables S1 and S2 [file mmc1.pdf]

iScience, Volume *28*

Supplemental information

Meningeal lymphatic architecture

and drainage dynamics surrounding

the human middle meningeal artery

Mehmet Albayram, Sutton B. Richmond, Kaan Yagmurlu, Ibrahim S. Tuna, Eda Karakaya, Hiranmayi Ravichandran, Fatih Tufan, Emal Lesha, Melike Mut, Filiz Bunyak, Yashar.S. Kalani, Adviye Ergul, Rachael D. Seidler, and Onder Albayram

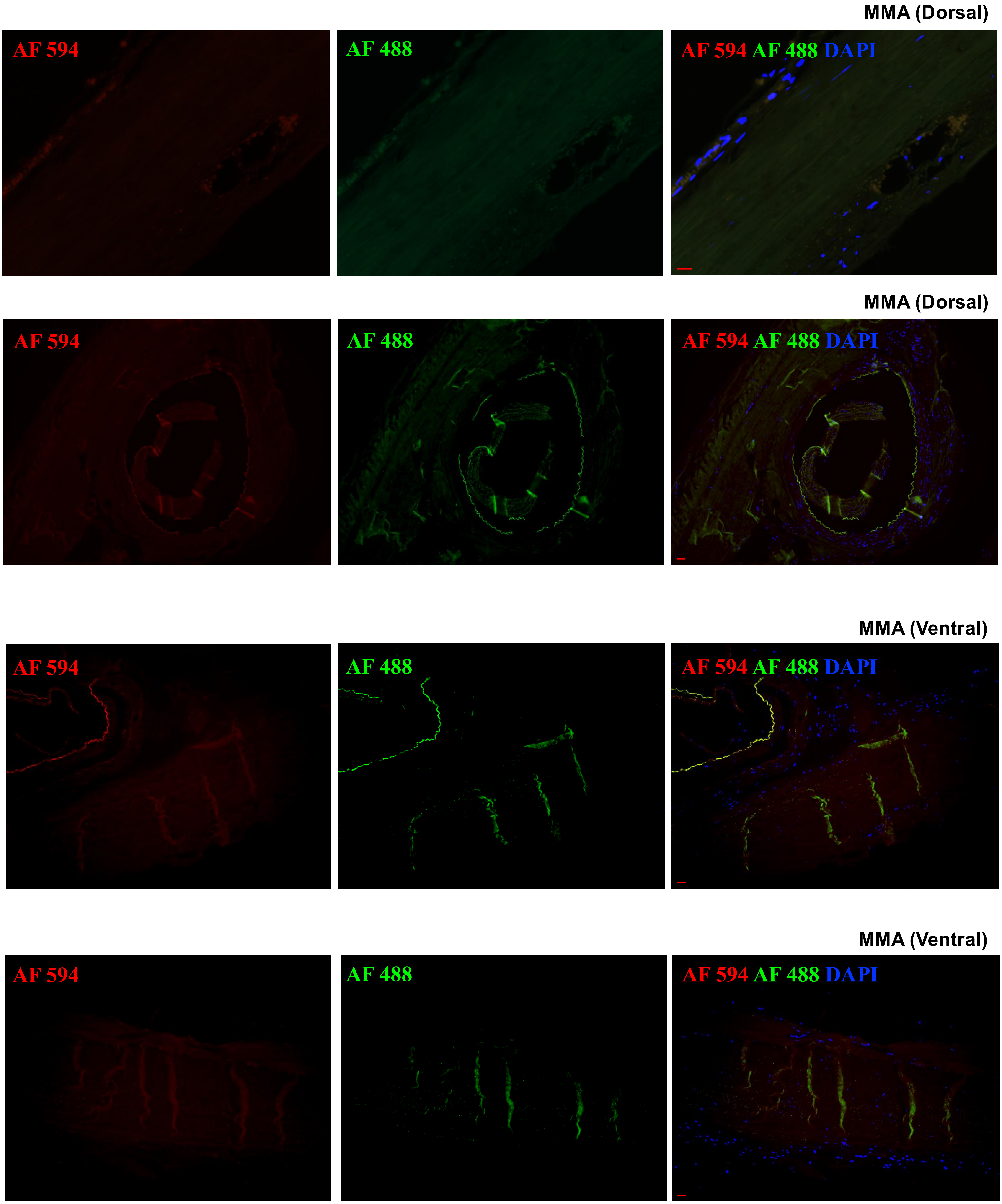

Figure S1: Negative control for immunofluorescence analysis of dorsal and ventral MMA dura. Dorsal and ventral segments of the human MMA were incubated with PBS in lieu of the primary antibody, followed by application of secondary antibodies. This approach acted as a negative control, allowing us to determine baseline fluorescence and assess any non-specific staining. Scale: 50μm. Figure S1 is related to main Figure 2.

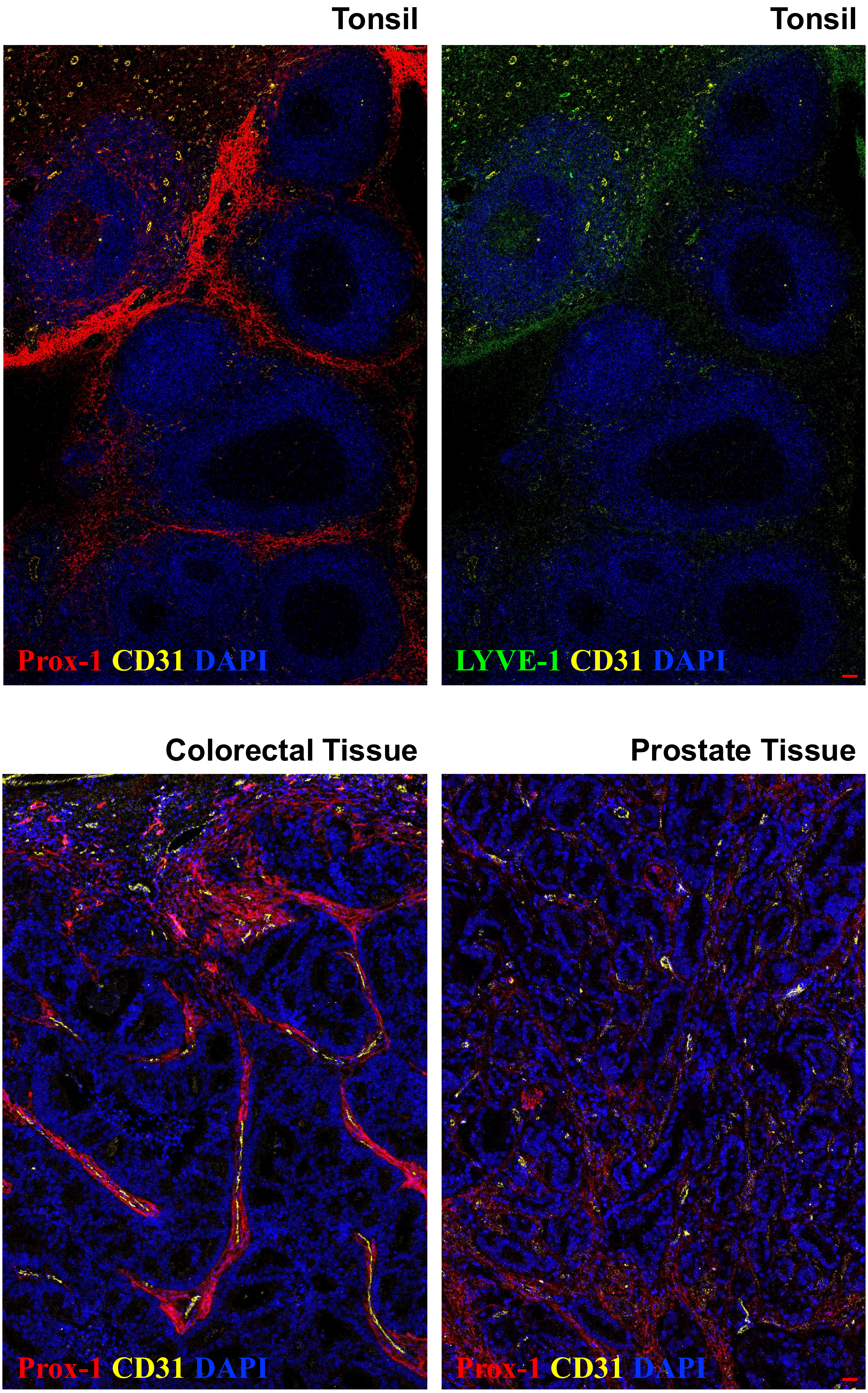

Figure S2: Positive control IMC staining of human peripheral tissues (tonsil, prostate, and colorectal) demonstrating lymphatic features. Prox1 (red) and LYVE1 (green) lymphatic signals, along with C31 (yellow), are present in IMC on peripheral tissues, including tonsils, prostate, and colorectal regions. Figure S3 is related to the main Figures 3 and 4. Scale, 50μm

A

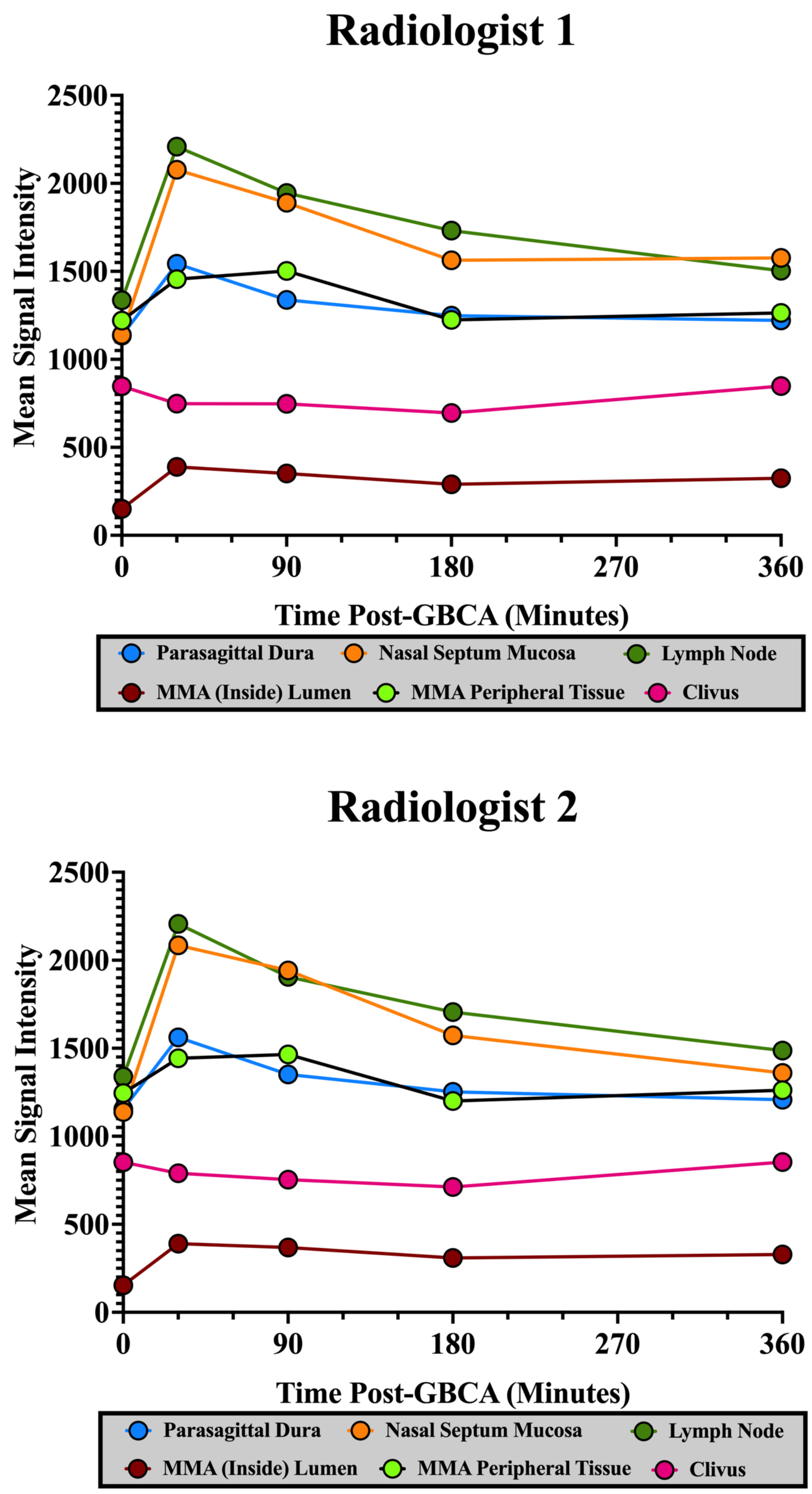

B

Inter-rater Agreement		
	**r (range)**	**p (range)**
**MMA-lumen**	0.97-0.99	0.001-0.006
**MMA-periphery**	0.98-1	<0.001-0.005
**Nasal Septum**	0.87-1	0.001-0.05
**Clivus**	0.97-1	<0:001-0.006
**Lymph Node**	0.94-1	<0.001-0.017
**Parasagittal Dura**	0.92-1	<0.001-0.028

Figure S3: Inter-rater validation of ROI-based MRI signal extraction across anatomical compartments. A) Mean signal intensity extractions from all six ROIs, across participants and time points, were executed by both neuroradiologists using the Picture Archiving and Communication Systems (PACS/ Visage 7.1.14, Visage Imaging®). **B)** Pearson correlation analysis revealed strong and positive correlations between the measurements of the two raters. Figure S3 is related to main Figure 1.

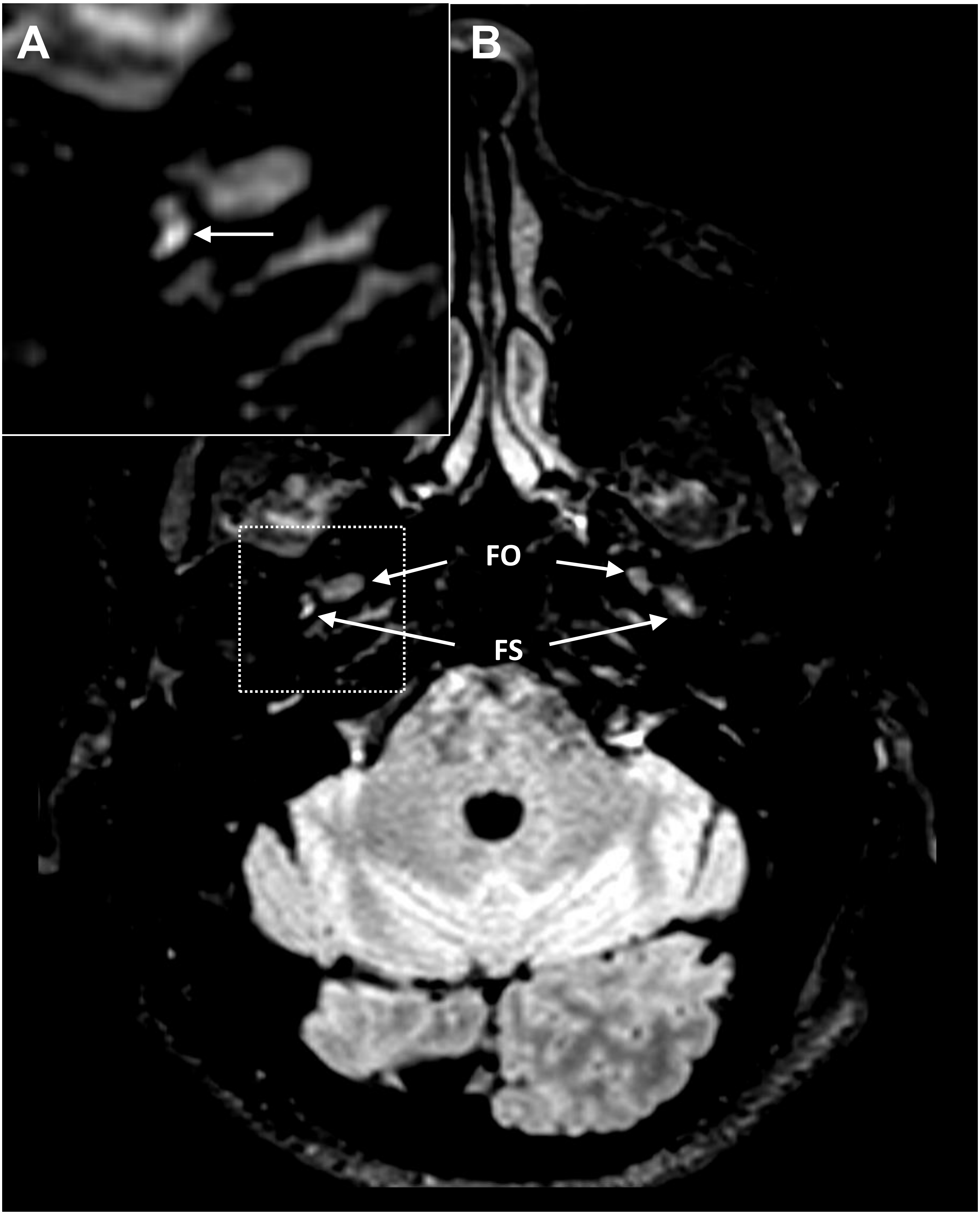

Figure S4: MRI visualization of the middle meningeal artery at the foramen spinosum and foramen ovale. Axial 3D fat-saturated FLAIR imaging of the brain from a 39-year-old male subject at the level of the middle cranial fossa **(B)**, with a magnified view **(A)** highlighting the regions of the foramen ovale (FO) and foramen spinosum (FS). A brighter signal is observed in the region of the middle meningeal artery (arrow in A). Figure S4 is related to STAR Methods.

Participant	Age (Years)	Height (m)	Weight (kg)	BMI (kg/m^2)	Sex	Race	Education
1	23	1.88	97.5	27.6	Female	Caucasian	Bachelor's degree
2	24	1.6	49.4	19.3	Female	Caucasian	Bachelor's degree
3	50	1.8	104.3	32.2	Male	Caucasian	Graduate school
4	21	1.55	54.4	22.6	Male	Caucasian	High School
5	28	1.8	65.8	20.3	Male	Caucasian	Bachelor's degree

Table S1: Individual MRI Participant Demographic and Anthropometric Summaries. This table summarizes age, sex, height, weight, body mass index (BMI), race, and the highest completed education level for each of the five healthy adult volunteers included in the final imaging analysis. All participants self-identified as Caucasian and were free of any medical contraindications for contrast-enhanced MRI. Education level reflects the participant’s highest attained degree at the time of enrollment. Table S1 is related to Figure 1

Average Mean Signal Intensity (*Normalized values to clivus)					
	TP_{Base}	TP_{30}	TP_{90}	TP_{180}	TP_{360}
**MMA-lumen**	151.9±23	389.5±64	359.7±63	299.7±66	326.1±52
**MMA-lumen***	0.191±0.04	0.535±0.12	0.518±0.13	0.419±0.08	0.396±0.07
**MMA-periphery**	1232.8±71	1449±151	1484±136	1212.3±44	1263.2±87
**MMA-periphery***	1.485±0.11	1.917±0.17	2.073±0.28	1.784±0.17	1.510±0.13
**Nasal Septum**	1138.3±59	2081.6±130	1916.3±79	1568.4±71	1467.9±220
**Nasal Septum***	1.384±0.13	2.781±024	2.653±0.24	2.281±016	1.752±0.29
**Lymph Node**	1337.2±135	2208±221	1925.7±104	1718.9±110	1495.1±75
**Lymph Node***	1.606±0.18	2.953±0.35	2.670±0.62	2.524±0.27	1.777±0.06
**Parasagittal Dura**	1144.9±58	1552.5±94	1344.6±114	1250.3±52	1214.5±144
**Parasagittal Dura***	1.413±0.18	2.120±0.25	1.947±0.38	1.826±0.15	1.501±0.26
**Clivus**	850±177.8	769±181.8	750±176.4	704±155.4	851±166.2

Table S2: Raw and normalized signal intensity (SI) values across anatomical regions and timepoints. This table presents the complete dataset of raw (non-normalized) signal intensity values for five anatomically defined regions of interest (ROIs)—MMA-lumen, MMA-peripheral, nasal septum mucosa, parasagittal dura (PSD), and deep cervical lymph node—collected at five timepoints (TP_0, TP_30, TP_90, TP_180, TP_360). Values were independently measured by two board-certified neuroradiologists in five healthy adult participants using T1-weighted contrast-enhanced MRI. Signal intensities for the clivus, used as the internal reference region, are also provided. Corresponding normalized values (ROI SI divided by clivus SI at the same timepoint) are included to demonstrate the full normalization pipeline. This resource enables transparent traceability of the quantification process and allows independent verification of spatiotemporal signal dynamics. Data represent mean±SEM. Table S2 is related to Figure 1.
